# Supplementary figures and images for: G protein subunit Gγ13-mediated signaling pathway is critical to the inflammation resolution and functional recovery of severely injured lungs
Source: eLife. 2024 Jun 5;12:RP92956. doi: 10.7554/eLife.92956 (PMC11152575; doi:10.7554/eLife.92956)

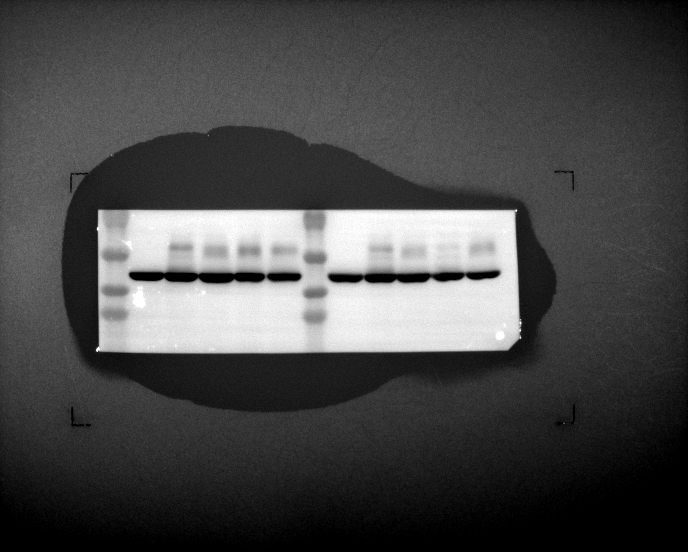

Supplement: Figure 4—source data 2. [file elife-92956-fig4-data2.zip › Figure 4-source data 2/Figure 4-source data 2-Figure 4D-╬▓-actin.tif]

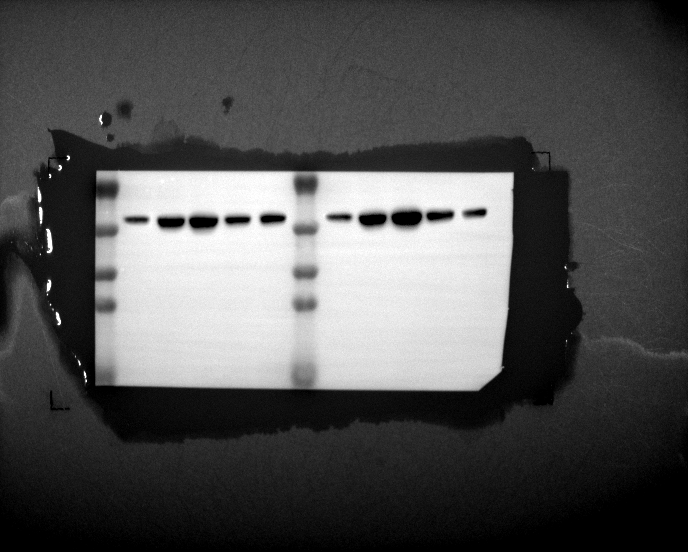

Supplement: Figure 4—source data 2. [file elife-92956-fig4-data2.zip › Figure 4-source data 2/Figure 4-source data 2-Figure 4D-Gsdmd-FL.tif]

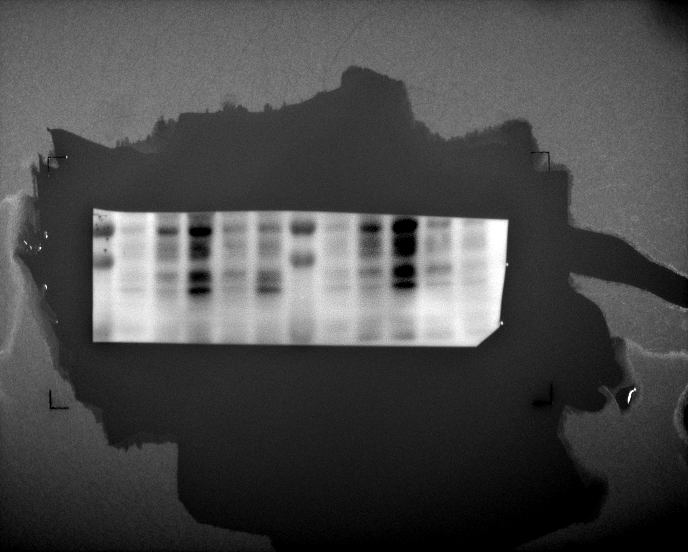

Supplement: Figure 4—source data 2. [file elife-92956-fig4-data2.zip › Figure 4-source data 2/Figure 4-source data 2-Figure 4D-Gsdmd-N.tif]

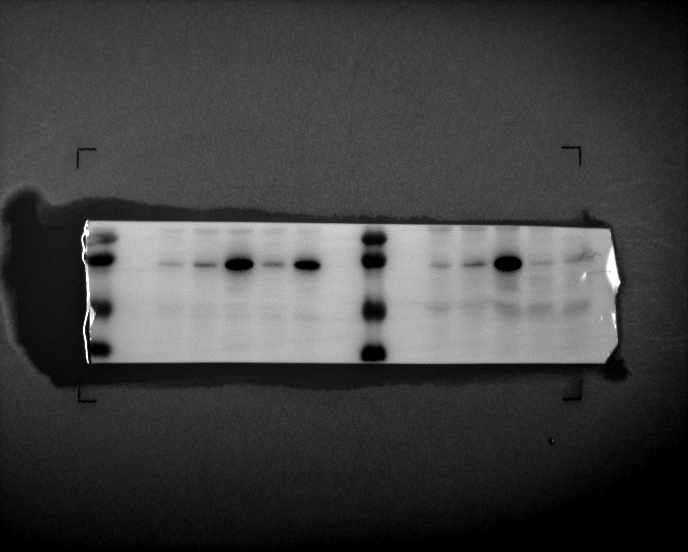

Supplement: Figure 4—source data 2. [file elife-92956-fig4-data2.zip › Figure 4-source data 2/Figure 4-source data 2-Figure 4D-IL-1╬▓.tif]

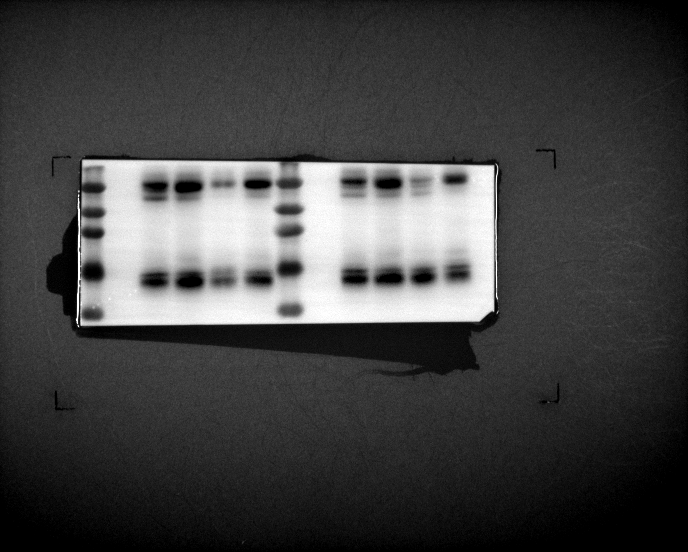

Supplement: Figure 4—source data 2. [file elife-92956-fig4-data2.zip › Figure 4-source data 2/Figure 4-source data 2-Figure 4D-Csap-1.tif]

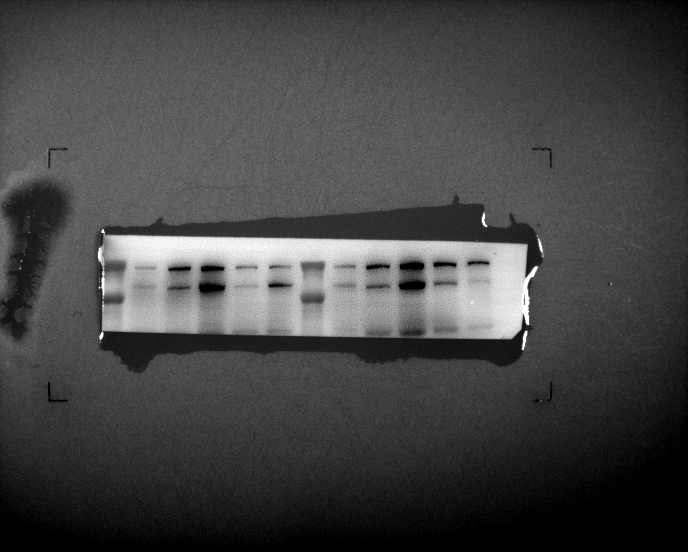

Supplement: Figure 4—source data 2. [file elife-92956-fig4-data2.zip › Figure 4-source data 2/Figure 4-source data 2-Figure 4D-NLRP3.tif]

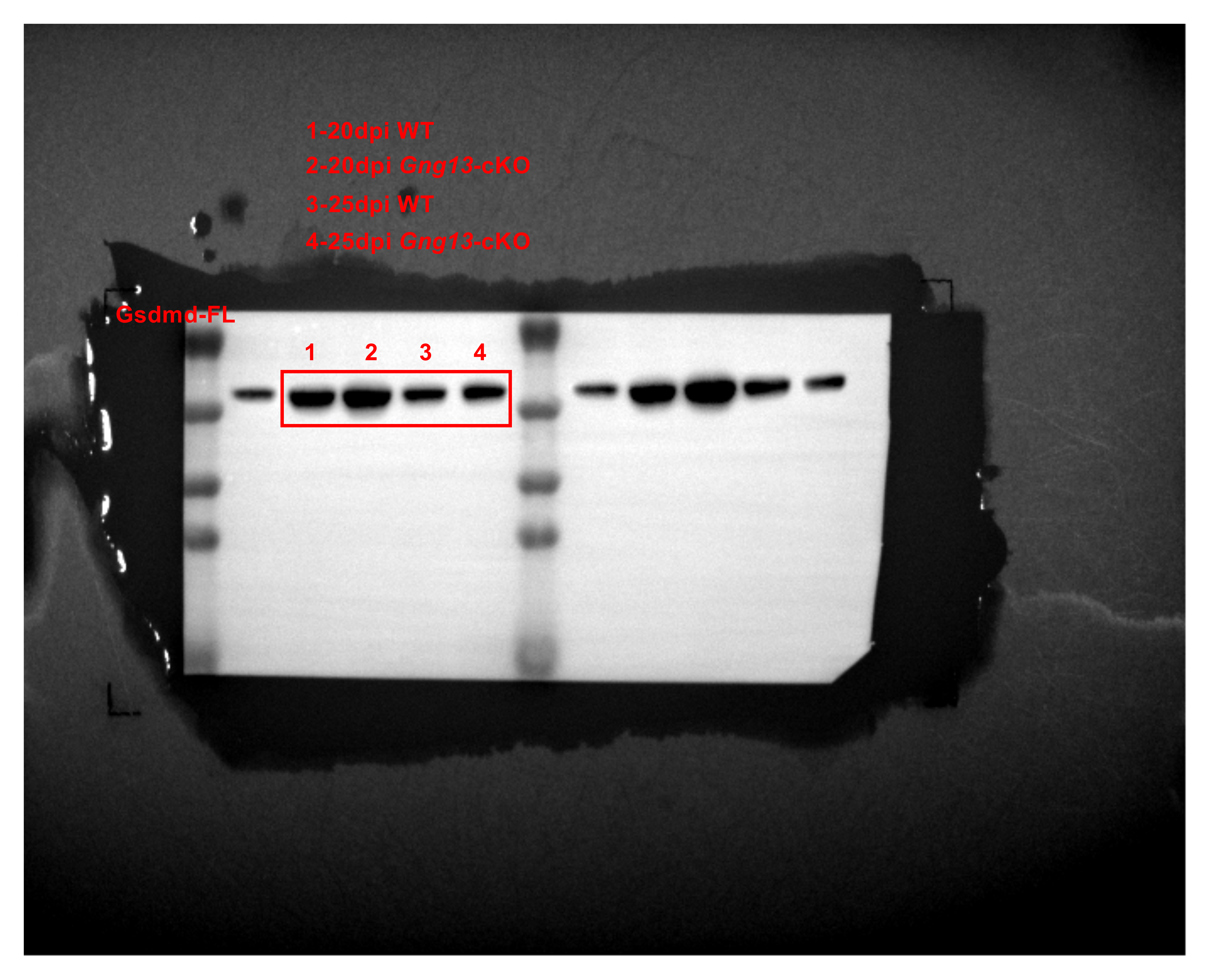

Supplement: Figure 4—source data 3. [file elife-92956-fig4-data3.zip › Figure 4-source data 3/Figure 4-source data 3-Figure 4D-Gsdmd-FL.tif]

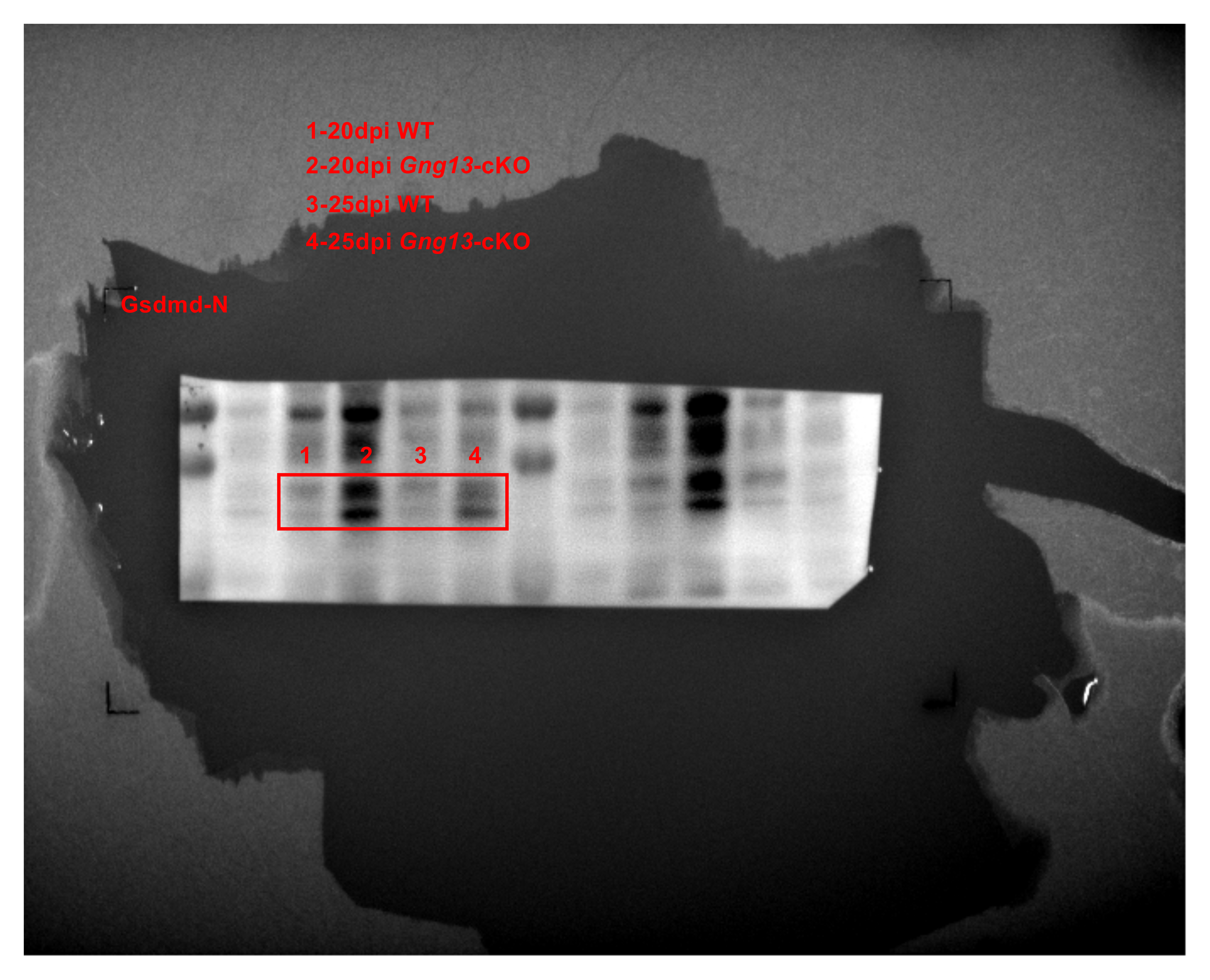

Supplement: Figure 4—source data 3. [file elife-92956-fig4-data3.zip › Figure 4-source data 3/Figure 4-source data 3-Figure 4D-Gsdmd-N.tif]

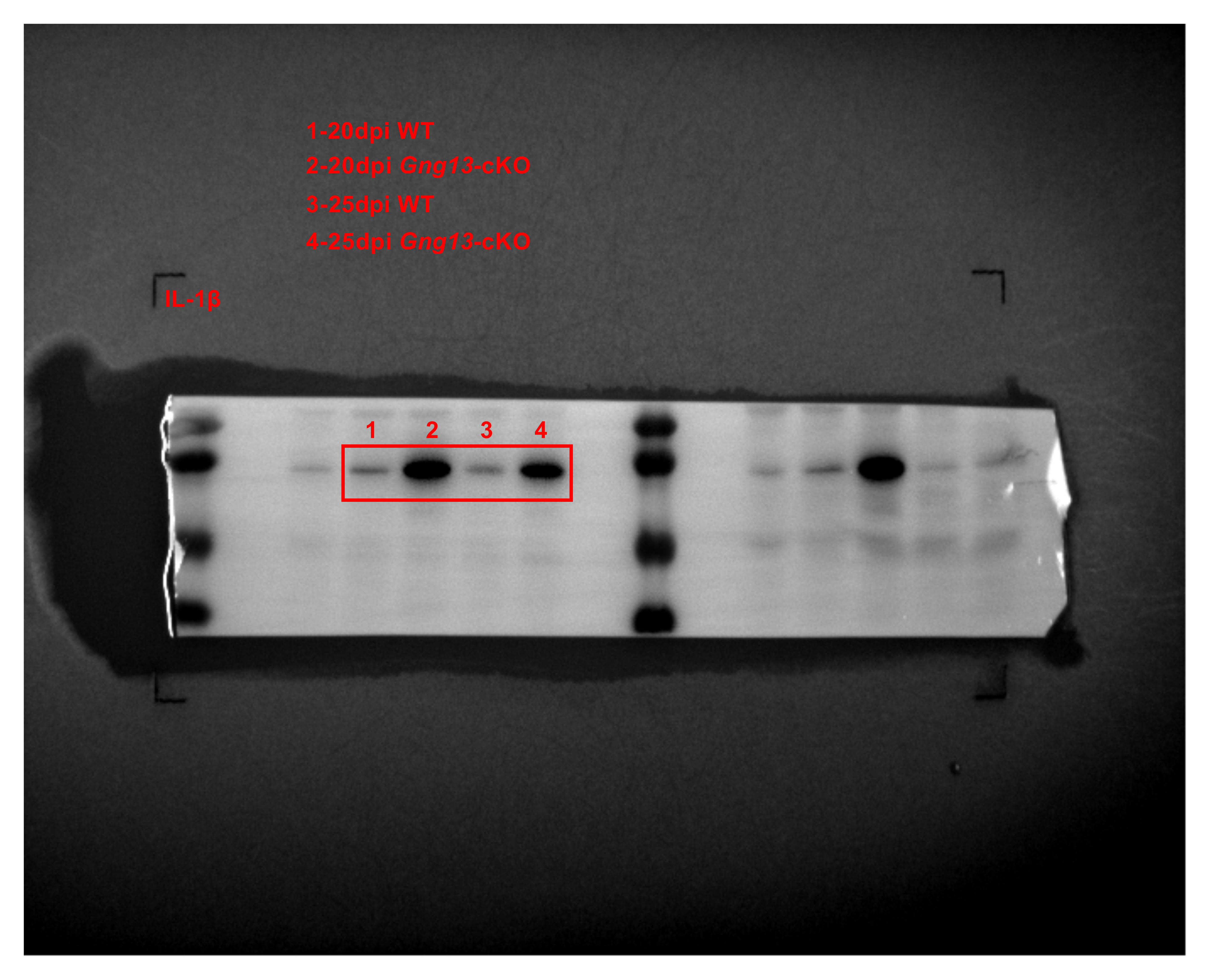

Supplement: Figure 4—source data 3. [file elife-92956-fig4-data3.zip › Figure 4-source data 3/Figure 4-source data 3-Figure 4D-IL-1╬▓.tif]

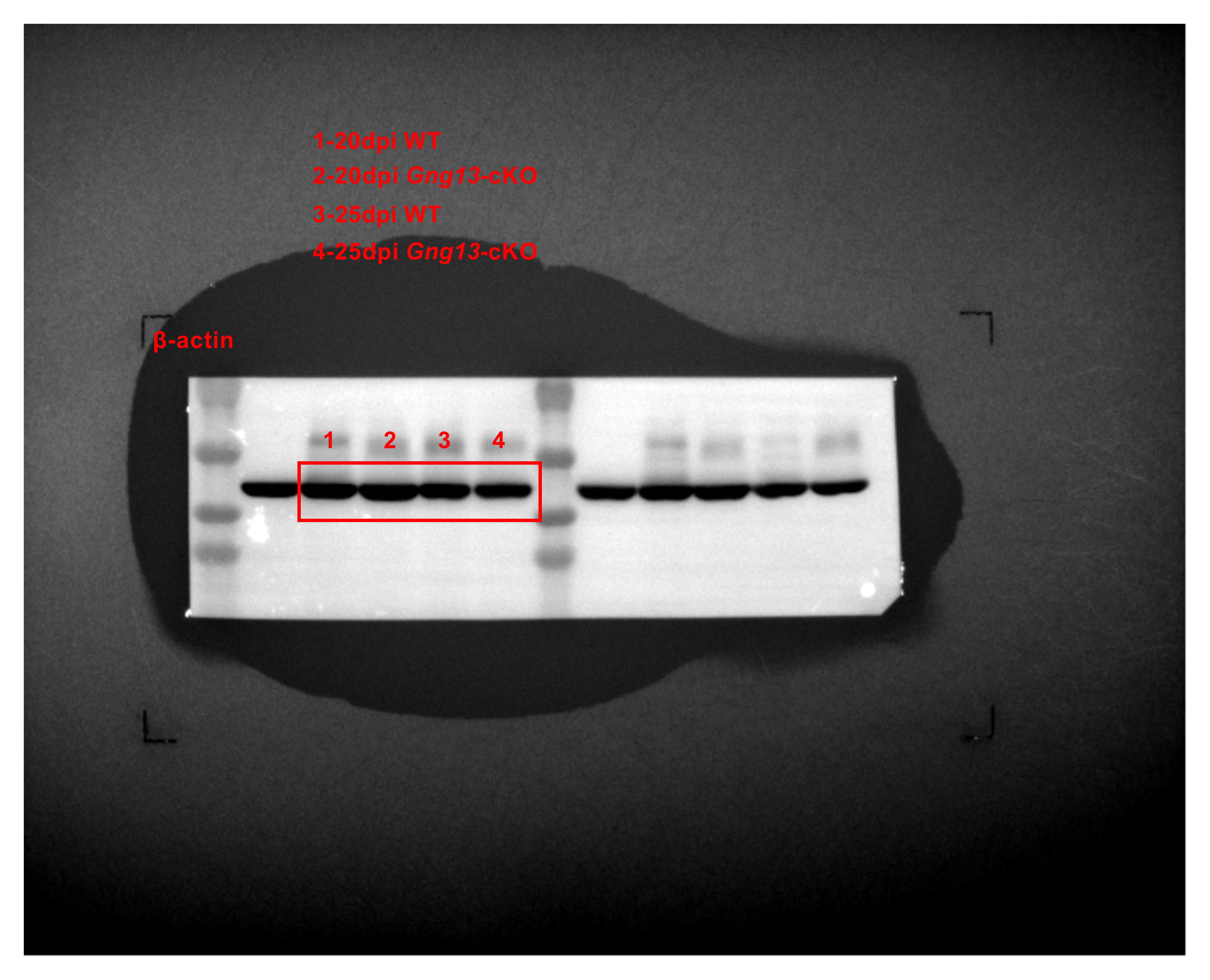

Supplement: Figure 4—source data 3. [file elife-92956-fig4-data3.zip › Figure 4-source data 3/Figure 4-source data 3-Figure 4D-╬▓-actin.tif]

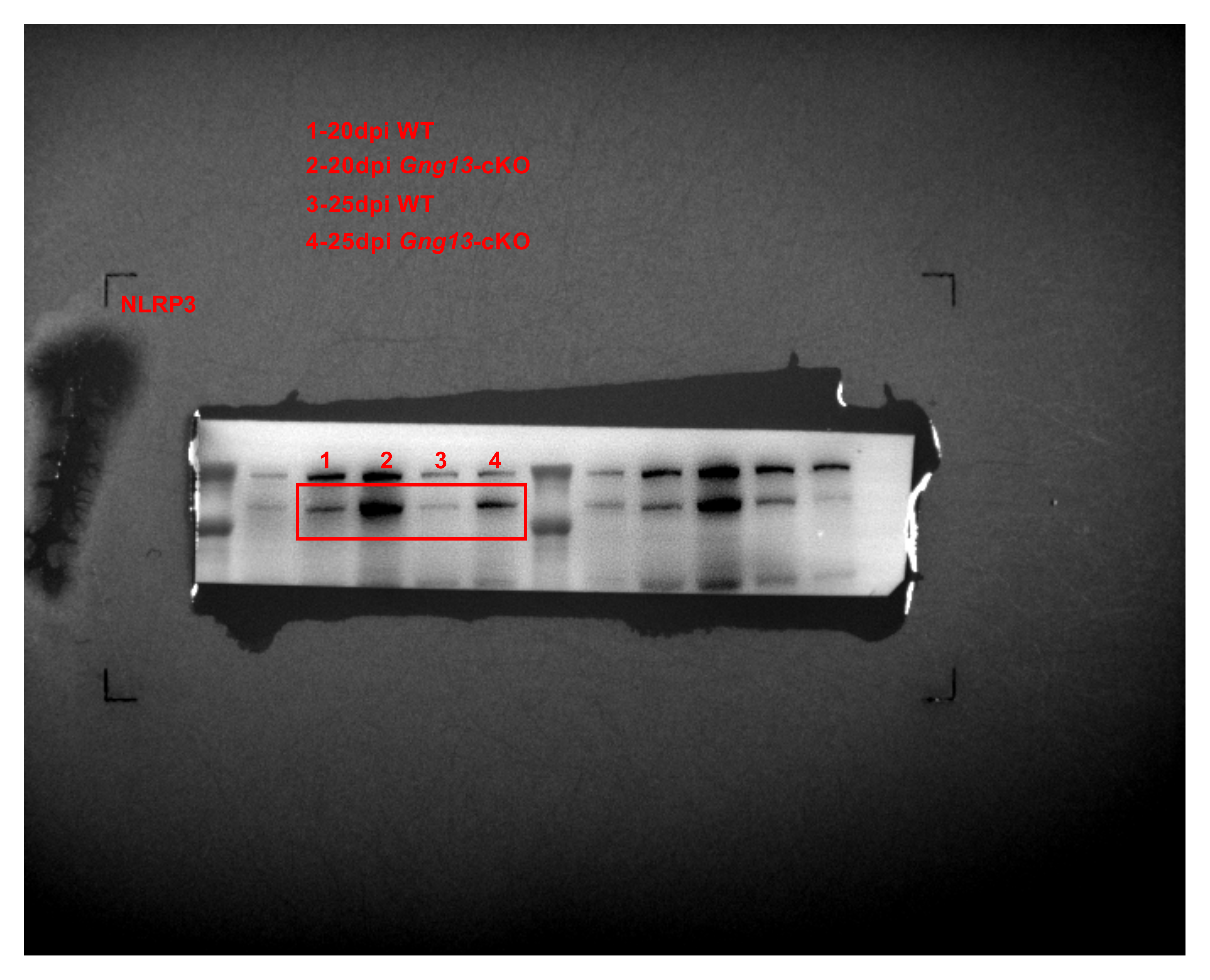

Supplement: Figure 4—source data 3. [file elife-92956-fig4-data3.zip › Figure 4-source data 3/Figure 4-source data 3-Figure 4D-NLRP3.tif]

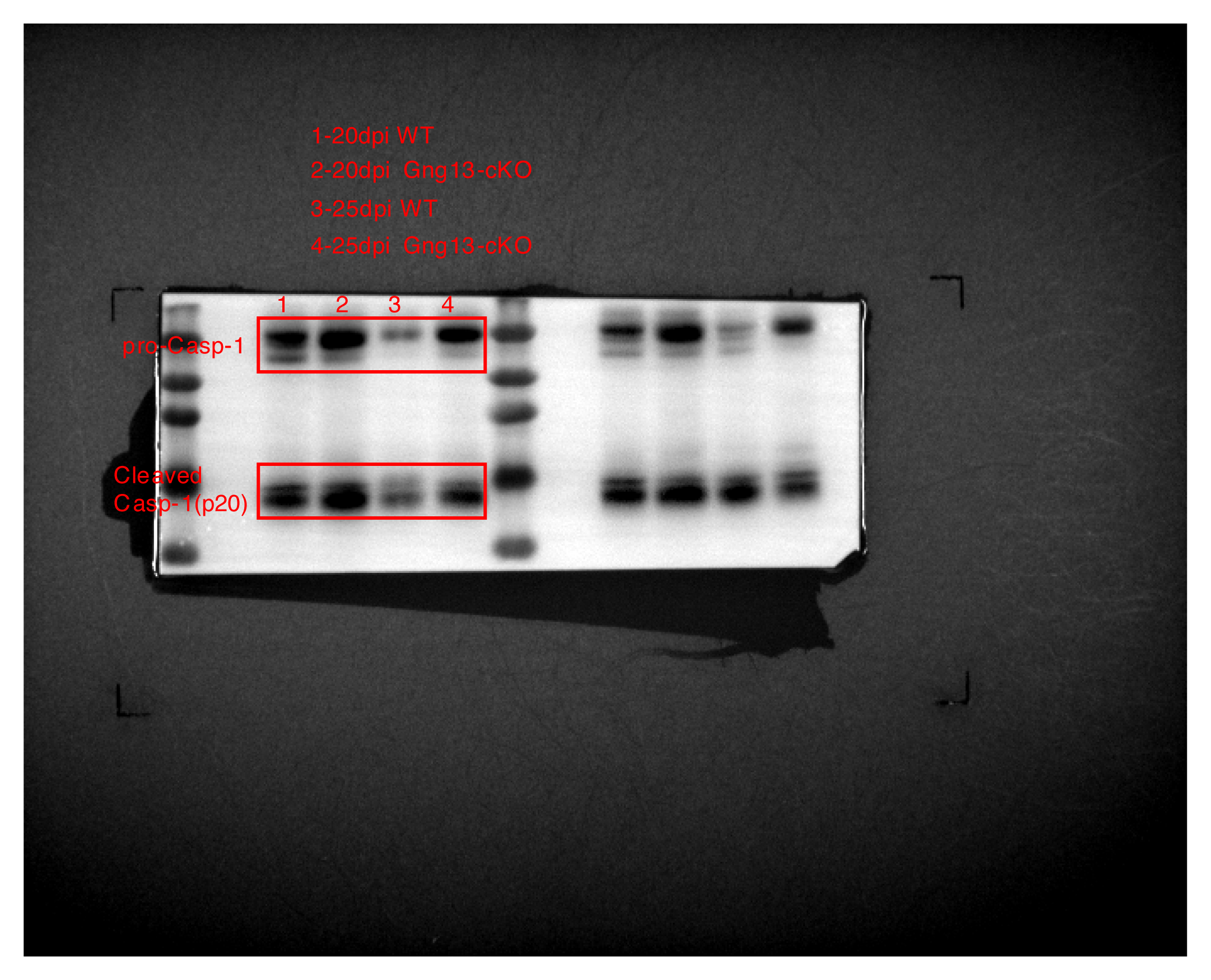

Supplement: Figure 4—source data 3. [file elife-92956-fig4-data3.zip › Figure 4-source data 3/Figure 4-source data 3-Figure 4D-Csap-1.tif]
